# Association of LRRK2 *rs11564258* single nucleotide polymorphisms with type and extent of gastrointestinal mycobiome in ulcerative colitis: a case–control study

**DOI:** 10.1186/s13099-021-00453-1

**Published:** 2021-09-30

**Authors:** Niusha Sharifinejad, Seyed Hamidreza Mozhgani, Mahmood Bakhtiyari, Elaheh Mahmoudi

**Affiliations:** 1 Student Research Committee, Alborz University of Medical Sciences, Karaj, Iran; 2 Alborz Office of USERN, Universal Scientific Education and Research Network (USERN), Alborz University of Medical Sciences, Karaj, Iran; 3 Department of Microbiology, School of Medicine, Alborz University of Medical Sciences, Karaj, Iran; 4 Non-Communicable Diseases Research Center, Alborz University of Medical Sciences, Karaj, Iran; 5 Department of Community Medicine, School of Medicine, Alborz University of Medical Sciences, Karaj, Iran; 6 Department of Mycology, School of Medicine, Alborz University of Medical Sciences, Karaj, Iran

**Keywords:** Inflammatory bowel diseases (IBD), Fungal microbiota, Dectin-1, Leucine-rich repeat kinase 2 (LRRK2), *rs11564258* polymorphism

## Abstract

**Background:**

Recently, the role of endogenous microbiota and the genotype-microbiota correlation in inflammatory bowel disease (IBD) pathogenesis have been highlighted. However, fungi, as the second most prevalent residents of the intestine, and their primary receptor, Dectin-1, are underrated. Thus, we conducted the first human study investigating the association of *Leucine-rich repeat kinase 2 (LRRK2)* polymorphism *(rs11564258)* with type and the extent of intestinal fungi in IBD patients.

**Material and methods:**

A case–control study was performed on 79 ulcerative colitis (UC)-patients (case group) and 58 healthy subjects (HS group). DNA was extracted from blood samples of both groups and amplified with the primers designed for the specific locus containing the LRRK2 polymorphism (*rs11564258*) and then sequenced. Dectin-1 and LRRK2 mRNA expression levels were also determined. Furthermore, the type and prevalence of fecal yeast species were surveyed in case and control groups.

**Results:**

A positive correlation was observed between *rs11564258* polymorphism and UC susceptibility (p = 0.008 vs. HS). Patients with active UC had the highest rate of isolated fungal colonies (50.41%), followed by patients with non-active UC (24.6%) and HS (25%). These results showed a relationship between UC severity with the increased fungal load. *Candida albicans* had the highest prevalence in both UC (78.7%) and HS groups (55.8%). Whereas *Saccharomyces cerevisiae* was the second most common species detected in HS (15.23%), it was significantly reduced in the UC patient group (1.68%) (*P* = 0.0001). On the other hand, single nucleotide polymorphism (SNP, *rs11564258*) was not correlated with the increased fungal flora in the UC patients. The expression of LRRK2 and Dectin-1 mRNA detected in blood samples was notably higher in the UC patients (*P* < 0.01) than in the HS group, without being affected by *rs11564258* polymorphism.

**Conclusions:**

Here, we disclosed that LRRK2 mediates Dectin-1 signaling pathway activation and subsequent inflammation in the UC patients without being affected by the presence of SNP *rs11564258*. Our data showed an increased global fungal load in the UC patients along with elevated UC susceptibility in cases carrying *rs11564258* polymorphism. However, more clinical investigations, particularly in larger populations with different ethnic groups, are required to support this conclusion.

## Introduction

The endogenous microbiota has an essential role in modulating the mucosal immune response in inflammatory bowel diseases (IBD), Crohn’s disease (CD), and ulcerative colitis (UC). The imbalance between the immune system and microbiota has been reported to cause IBD in genetically prone individuals [1–3]. Although the exact role of fungal colonization and their diversity has not been precisely defined in the pathophysiology of IBD, the increased fungal richness and diversity, as well as shifting in the fungal spectrum, were determined in patients with IBD. This observation is contrary to the enteric bacterial community [4].

Of note, a majority of single-nucleotide polymorphisms (SNPs) that modulate the risk of genetic susceptibility to IBD are involved in immune responses, which highlights the importance of immune signaling in IBD development. Dectin-1 is the most important intestinal fungal pattern recognition receptor (PRR) expressed by the innate immune cells and has an established role in severe forms of colitis [5]. *C-Type Lectin domain containing 7A (CLEC7A)* is the gene that directly encodes Dectin-1 and has been associated with colitis [6, 7]. Other than *CLEC7A*, *leucine-rich repeat kinase 2 (LRRK2)* is another gene polymorphism that influences Dectin-1-associated immunity in IBD [8, 9]. The SNP *rs11564258* at the locus of the LRRK2/MUC19 gene region creates a high-risk genetic locus for the development of IBD via dectin-1 signaling. Mice with a heterozygous mutation at this site exhibited more severe colitis than their wild-type littermates [10]. LRRK2 is a large multi-domain protein with dual GTPase and kinase activities. This protein is highly expressed in immune cells and has been functionally linked to pathways pertinent to immune cell function, such as cytokine release, autophagy, and phagocytosis [9]. Moreover, it induces an increase in the activation of the intestinal dendritic cells (DCs), which leads to an amplified expression and release of pro-inflammatory molecules such as *tumor necrosis factor-alpha (TNF-α)* associated with IBD [10, 11]. *LRRK2* inhibitors decreased Dectin-1-induced *TNF-α* production by mouse DCs and ameliorated colitis, both in control and *LRRK2* transgenic animals [10]. Furthermore, LRRK2 takes part in the control of the DCs immune response to *Aspergillus* through a non-canonical autophagic response of DCs to the germinated spores of this fungus [12]. Therefore, understanding the reciprocal interaction between the host immune system and the intestinal mycobiome is of great biomedical importance. To investigate the correlation between the *LRRK2* polymorphism with intestinal fungal abundance and diversity in the UC patients, we analyzed the *LRRK2* polymorphism and the fecal fungal community of the UC patients. We also aimed to highlight the role of the genotype-mycobiome association in IBD pathogenesis.

## Materials and methods

### Patients and samples collection

A total of 137 individuals were assessed, composed of 79 UC patients and 58 healthy subjects (HS). All patients were recruited at the Gastroenterology Department of the Emam Ali Hospital (Karaj, Iran) and provided informed consent. UC was diagnosed according to clinical, endoscopic, and histological criteria. None of the participants had taken corticosteroids, antibiotics, antifungals, or used colon-cleansing and probiotic products for at least 2 months before enrolment. We also excluded patients with other chronic diseases. Samples including peripheral blood, tissue, and fecal were collected from patients with UC and HS. Given the low prevalence of patients with CD and to achieve an accurate result, we excluded CD patients in the following comparisons.

### Ethics

The patients or their companions gave their written informed consent before inclusion in the study. The local ethical committee of Alborz University of medical sciences approved the study No IR.ABZUMS.REC.1398.070, which conforms to the guidelines of the Declaration of Helsinki.

### Histological assessment

Paraffin-embedded colon tissues were sectioned and stained with H&E for pathology assessment. Colitis severity assessment was conducted using the disease activity index (DAI), scoring from 0 to 4, according to criteria such as increased inflammation, erosion, crypt abscess, crypt atrophy, goblet cell depletion, fibrosis, and granuloma. The total histological score was given as epithelium plus infiltration.

### Genotyping

For genotyping of SNP *rs11564258*, we used a PCR–Sanger sequencing assay. We took 2-ml whole blood samples of the patient and healthy groups and poured them into EDTA tubes. These samples subsequently underwent DNA extraction using Kit (DNSol Maxi Kit, Roje-Technologies) as per manufacturer̕ s instructions. The quantity and quality of DNA samples were assessed by NanoDrop 2000c (Boeco, Germany). Polymerase Chain Reaction (PCR) was performed in reactions containing 13 μl of 2X ready to use Master Mix (SinaClon, Iran), 1 μl of each 20 pmol forward and reverse primers, 8 μl of sterile distilled water, and 2 μl extracted DNA template. The PCR thermal conditions were as follows: 94 °C (5 min), {95 °C (40 s), 62 °C (40 s), 72 °C (20 s)} × 45, 72 °C (5 min). Electrophoresis using 1% agarose gel was performed to evaluate PCR amplicons. Sequencing analysis was performed on purified PCR products using the forward primer on the Applied Biosystems 3730 XL DNA Analyzer instrument (Bioneer Corporation, Korea). The sequences were compared with the GenBank database (http://www.ncbi.nlm.nih.gov), using the Blast system. The primer sequences are mentioned in Table 1.

**Table 1:** Sequences of primers used in this study

Target	Primer (5ʹ to 3ʹ)	Amplicon sizes (bp)	TM (°C)
LRRK2( DNA) (SNP *Rs11564258*)	F:ACCAAGGATACCCTAACTTCTTACCAA R: TCGATGGTGTTCTCAGCCCA	330	62
LRRK2 (mRNA)	F:GGGTGCGAAGAGGACGAGGA R: AACCCACCTGCTGCACTC	223	62
Dectin-1	F:GATTTAGAAAATTTGGATGAAGATGG R:TATCACCAGTATTACCAAGCATA	165	59
GAPDH	F: ATGCCTCCTGCACCACCAAC R: TGACCTTGCCCACAGCCTTG	216	60

### Analysis of gene expression by real-time PCR

Dectin-1 (Clec7a), a pattern recognition receptor expressed by intestinal epithelial cells, and its associated enhancer (LRRK2) are under investigation for their close relation to intestinal inflammation & colitis. In order to measure their expression, total RNA of whole blood from UC patients and HS was extracted using the high pure RNA isolation kit (Roche, Switzerland) according to the manufacture’s instruction. The quality and quantity of RNA concentrations were analyzed by NanoDrop 2000c (Boeco, Germany). According to the kit protocol, cDNA was synthesized from 1 μg of RNA using Transcriptase First Strand cDNA Synthesis Kit (Roche, Switzerland). cDNA was adjusted as the same concentration for all samples (1μL of 1 μg adjusted cDNA). The primer sequences and melting temperature used in PCR are presented in Table 1. Relative real-time PCR was determined in double reaction in final volume up to 20 µl including 13 µl of 2X ready to use SYBR Green Master Mix (Amplicon-Denmark), 1 μl of each 20 pmol forward and reverse primer with 1 μl adjusted cDNA(1 μg/1μL), 4 μl nuclease-free water. The RT-qPCR thermal conditions were as follows: Hold1: 94 °C (6 min), Cycling: {94 °C (45 s), 62 °C/59 °C (LLRK2/Dectin1) (35 s), 72 °C (30 s)} × 35, Hold2: 30 °C (2 min). Quantification and analysis were carried out in Rotor-Gene Q 5plex (Qiagen, USA). The GAPDH gene was amplified as an internal control to normalize the mRNA expression of the target genes. The differences in gene expression were calculated using the 2-ΔΔCt method [13], in which ΔCt indicates the difference of Ct values of the target gene and GAPDH.

### Fungal analysis in healthy subjects and patients with UC

Whole stools were collected in sterile boxes, and 1 g of stool was resuspended in 1 ml of physiological saline for further analysis. Microscopic identification was performed on a wet mount direct potassium hydroxide (KOH) preparation for the detection of fungal elements. To isolate the fungal species and ensure about detection of all yeast and mold colonies, 10 μl of each sample were incubated for 3–5 days at 37 °C in Sabaro dextrose agar (Sigma, Germany) and Corn Meal Agar with Tween 80, supplemented by chloramphenicol (0.05%), respectively. For specific analysis of *C. albicans*, 10 μl of each sample was inoculated on chromogenic *Candida* agar (bioMerieux, France). Furthermore, *Candida* species were differentially diagnosed based on the germ tube test and attendance of catalase.

### Molecular identification

DNA of isolated fungal cultivable single-cell pure colonies was extracted using the phenol–chloroform protocol described by Yamada et al*.* [14]. PCR was then used to amplify the ITS-5.8S rDNA region using the universal primers ITS4 F: 5΄-GGAAGTAAAAGTCGTAACAAGG-3΄and ITS5 R: 5΄ TCCTCCGCTTATTGATATGC-3΄ [15]. All PCR-amplified products were sequenced using the Applied Biosystems 3730 XL (Bioneer Corporation, Korea) and ITS4 primer. Sequence search was performed through local blast with a molecular database maintained at the NCBI (Library of Medicine, Bethesda, MD, USA; http://www.ncbi.nlm.nih.gov/BLAST/).

### Statistical analysis

The significance of the results in this study was assessed with odds ratios derived from a binary logistic regression model. Analysis of variations in the distribution of each of the examined species between the study groups was analyzed using one-way ANOVA and Tukey post hoc multiple comparisons whereas differential gene expression for the studied genes was determined using Kruskal–wallis test with Dune’s multiple comparisons and Non-parametric Mann–Whitney U-test. Pairwise comparison between the groups for the remaining continuous and categorical variables was achieved using Independent T-test and Pearson chi-square test, respectively. A *P*-value of less than 0.05 in tests was considered statistically significant. All of the statistical analyses were performed using SPSS version 24.0 (IBM, New York, USA).

## Results

### Distribution of the LRRK2 SNP (rs11564258) in UC patients and HS

Among the alleles in the LRRK2 gene locus, the risk allele at *rs11564258* located downstream of LRRK2 has the highest risk polymorphism for IBD and heterozygote animal cases exhibited increased severity of colitis. Given the low prevalence of patients with CD, we excluded CD patients in this study. Our results showed a significant genotypic distribution difference at SNP rs11564258 between the UC and HS cohorts. Specifically, as shown in Table 2, the GA heterozygous variant genotype was more likely to was occur in the UC patients (36.7%, 29/79) than in the healthy subjects (15.5%, 9/58) (OR 3.1; 95% CI [1.36–7.35]; P = 0.008). Only one UC patient, but not in any of the healthy controls, was shown to carry the AA homozygous variant genotype.

**Table 2:** Comparison of *rs11564258* [n(%)] SNP and genotype between UC patients and HS

Genotype	UC, n (%) (n = 79)	HS, n (%) (n = 58)	P-value	Odds ratio (OR)	95% Confidence interval
GA	29 (36.7%)	9 (15.5%)	0.008	3.1	1.36–7.35
AA	1 (1.26%)	–	–	–	–
GG	49 (62.04%)	49 (84.5%)	–	–	–

### Correlation of the rs11564258 SNP with the severity of UC

**Fig. 1:**
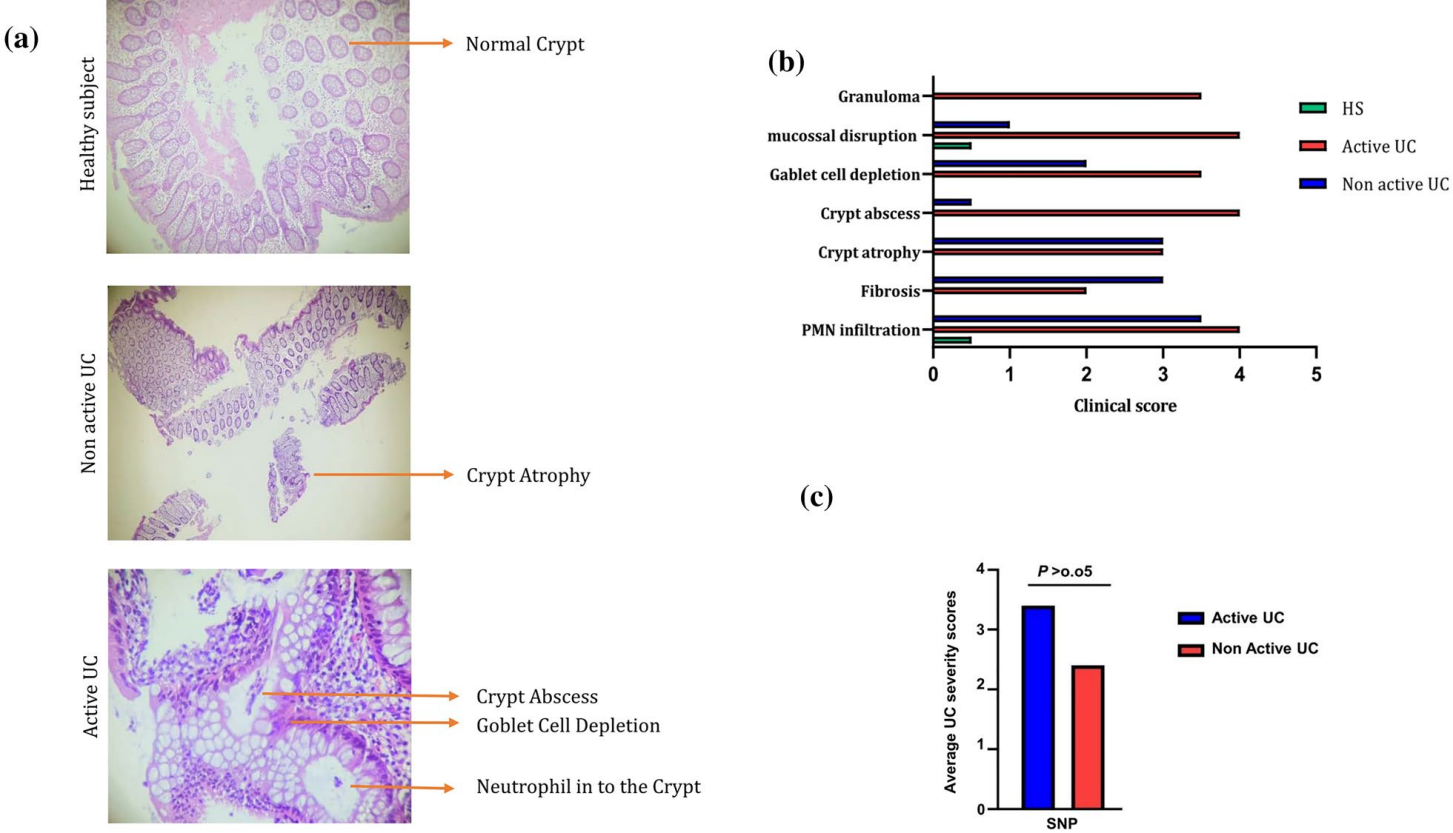
**a** H&E stained colon sections of HS, non-active UC, and active UC samples. The picture was taken using an Olympus D330 digital camera (Olympus, Tokyo, Japan) (×40 magnifications). **b** Damage scores ranged from 0 to 4, as described in the text. Scales were judged based on the number and extent of PMN infiltration, mucosal disruption, crypt abscess, crypt atrophy, goblet cell depletion, fibrosis, and granuloma. **c** There was no significant difference between the average UC severity scores of the SNP-positive and SNP-negative patients in the UC group. Data are presented as mean ± SD

The colonic tissue specimen was used for histologic confirmation and severity evaluation of UC. Based on the pathology evaluation of tissue sections, our study population was divided into healthy subjects, non-active UC, and active UC groups. The active UC group had the highest clinical score of tissue damage and inflammation among all the examined groups (Fig. 1a and b). The UC patients carrying SNP rs11564258 were compared to patients with the wild-type genotype G/G. Evaluations of genotype–phenotype correlations did not reveal a significant association between genotype and a specific phenotype such as age at diagnosis, gender, family history of IBD, and recurrence of the disease (Table 3). Furthermore, this SNP was not correlated with UC severity and flares in the affected patients (Fig. 1c, *P* > 0.05).

**Table 3:** Genotype–phenotype correlation of demographic characteristics of the patients

Parameters	G/G genotype (n = 98)	G/A genotype (n = 38)	A/A genotype (n = 1)	P-value
Age of onset, median (IQR), y (n = 137)	48.0 (28.8–62.0)	43.6 (31.1–55.4)	35.0	0.064
Male/Female ratio (n = 137)	42/55	17/22	0/1	1.000
Family history of IBD (%) (n = 137)	22 (22.4)	9 (23.6)	1 (100)	0.323
Disease recurrence (%) (n = 137)	40 (40.8)	16 (42.1)	1 (100)	0.675

### LRRK2 SNP (rs11564258) did not affect gene expression of dectin1 and lrrk2

**Fig. 2:**
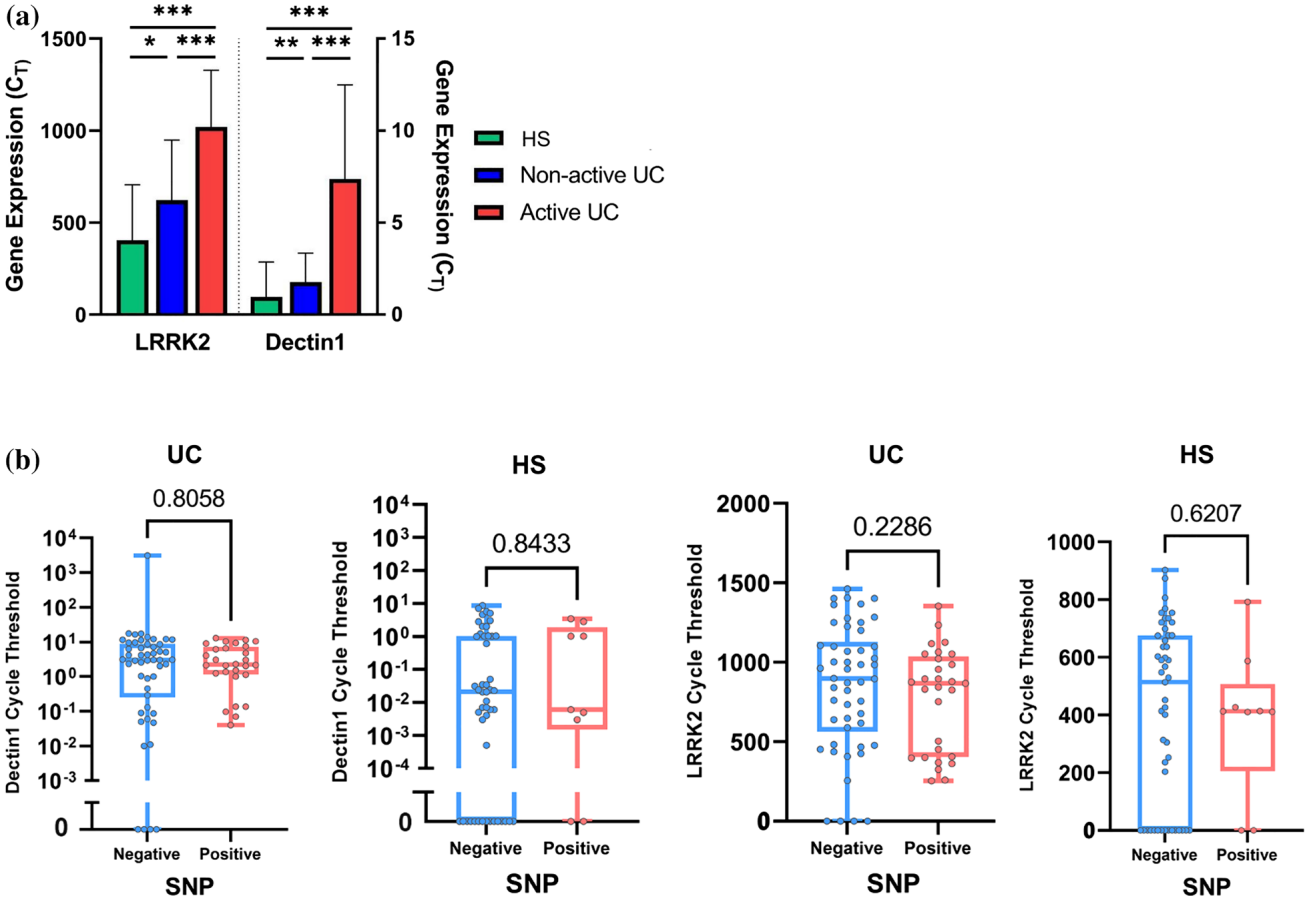
**a** Relative change of Dectin-1 and LRRK2 mRNA level in HS, non-active UC, and active UC. (*P = 0.01), (** *P* = 0.001) (***P = 0.0001). **b** SNP (*rs11564258*) did not affect gene expression level of Dectin-1 and LRRK2 mRNA in all groups (p > 0.05)

To assess whether the LRRK2 polymorphism affects Dectin-1 and LRRK2 mRNA expression, the relative expression of CLEC7A and LRRK2 genes was further determined by real-time PCR. The expression level of these genes was significantly different between the UC patients and HS, with a higher expression in the active UC group (*P* = 0.0001, Fig. 2a). However, there was no remarkable gene expression difference in either gene between the SNP-positive and SNP-negative samples in each UC and HS group (Fig. 2b, *P* > 0.05).

### Altered fungal mycobiota diversity in UC patients

**Fig. 3:**
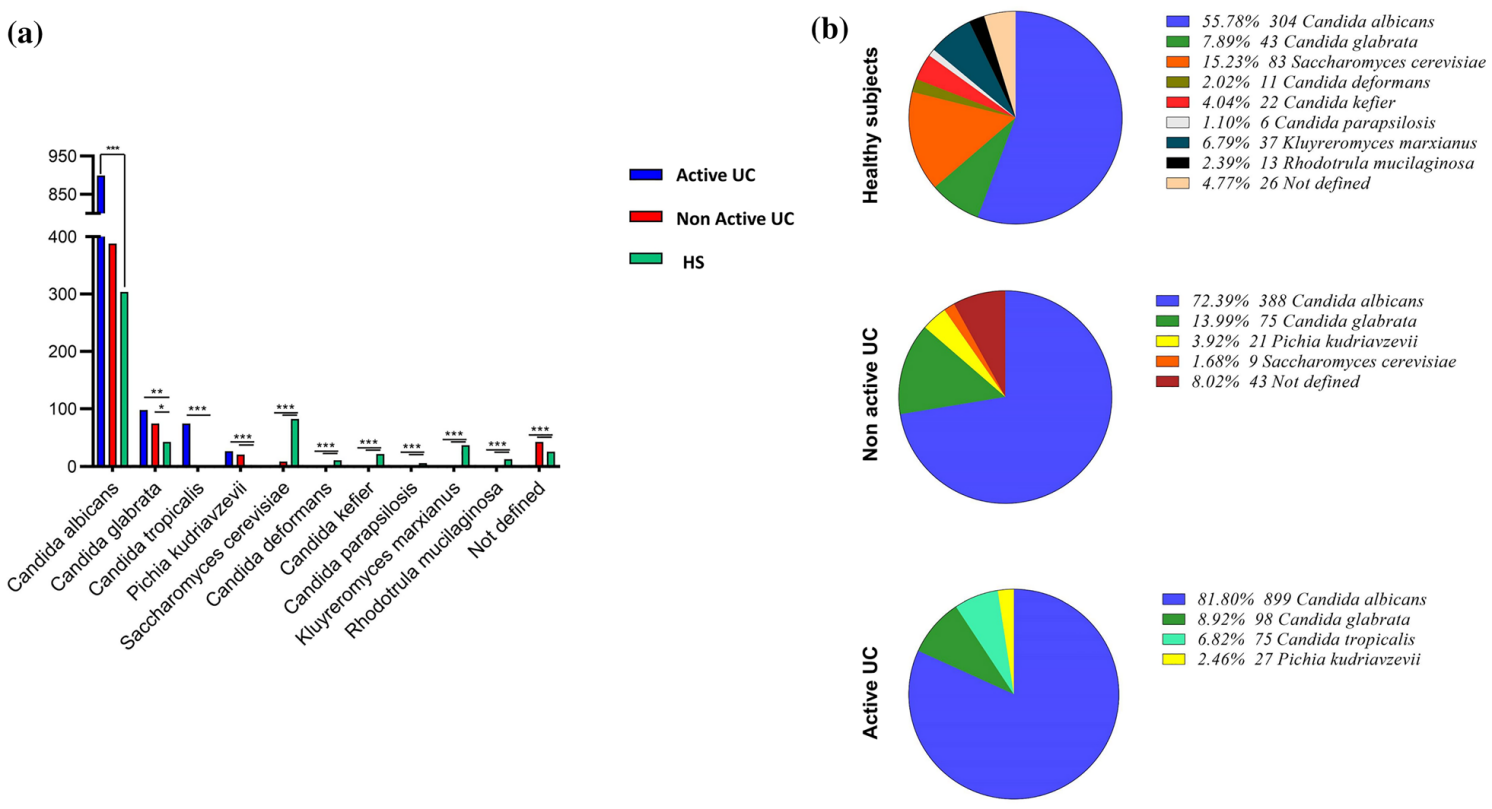
Frequency of fungal species isolated from UC patients (active and non active) and HS. Fungal composition was determined using ITS2 sequencing. Our results showed the **a** CFU abundance (per gram) and **b** relative abundances (%) of fecal mycobiota in patients with UC and HS. Data are presented as mean ± SD. (*P = 0.01), (**P = 0.001) (***P = 0.0001)

Using macroscopic differentiating methods and ITS sequencing, we assessed the composition of the intestinal fungal microbiota in our population. Among the fungal colonies (CFU/g) isolated from fecal samples, *Candida albicans* (70%), followed by *Candida glabrata* (10.3%), and *Saccharomyces cerevisiae* (8.4%) were the most prevalent yeast species. UC patients had the highest rate of isolated fungal colonies (75%) compared with the HS (25%) (Fig. 3a). On the other hand, the intestinal mycobiota of the HS group showed greater variability than UC (Fig. 3b). While *C. albicans* was the most predominant fungal species isolated from both UC (78.7%) and HS groups (55.8%), Saccharomyces cerevisiae, the second most common species detected in HS (15.23%), was significantly less abundant in the UC patients (1.68%) (Fig. 3a, *P* = 0.0001). *C. glabrata* was the second most abundant species isolated from UC samples, followed by *C. tropicalis*. These data showed the specific alterations in fungal microbiota diversity and a positive correlation with the severity of UC (Fig. 3a). Notably, LRRK2 SNP (*rs11564258*) was not correlated with the abundance of fungal flora in UC patients (data not shown).). In addition, we exclusively found *Candida deformans*, *Candida kefier*, *Candida parapsilosis, Rhodotorula* and *Kluyreromyces* genera, in the HS (Fig. 3b).

## Discussion

IBD is assumed to derive from an improper inflammatory response to intestinal microbes in a genetically susceptible host. Despite extensive bacterial studies in IBD, the role of intestinal fungal flora is underrated [7]. In this study, we investigated the association of *LRRK2 (rs11564258)* polymorphism with IBD susceptibility and severity along with the type and extent of intestinal fungi obtained from fecal samples of IBD patients. However, due to the higher prevalence of UC in our patients and the overall Iranian population [16] compared to other nations [17], we excluded CD patients in our results. Our data revealed a significant association between *rs11564258* mutation and UC susceptibility. Contrary to the murine study of Takagawa et al. [10], the UC severity and flares did not notably differ among the patients bearing this variance (data not shown). As opposed to some previous studies [18–20], we did not detect a significant increase in the global fungal load in the UC patients carrying *rs11564258* mutation. Furthermore, the odds ratio of this variant *(rs11564258*) was 3.1 in UC patients, albeit this polymorphism also confers an increased CD risk (OR = 1.74) [21].

The global fungal richness of the UC patients in the active phase (50.41%) was greater than the HS (25%) and UC patients in the non-active phase (24.6%), respectively, with *C. albicans* as the dominant species in the UC cases. Similarly, another study reported an increased global fungal load in both inflamed and non-inflamed mucosa of the IBD patients compared to healthy controls [22]. A close prevalence was also observed in the familial CD patients [23, 24]. These results show the relationship between the UC severity and increased fungal load. Also, they highlighted the critical role of *C. albicans* in the pathophysiology of UC. As formerly reported by various articles, *S. cerevisiae* tends to be decreased in IBD, especially during CD [25–27]. This species had a higher prevalence in our control group compared to the UC group. Moreover, Di Paola et al*.* assumed that the absence of *S. cerevisiae* was related to a potentially pathogenic bacteria that could lead to IBD [28]. In contrast to other reports [27, 29, 30], the proportions of *Ascomycota* and *Basidiomycota* in our samples were not significantly different between the studied groups. We also reported *Pichia kudriavzevii,* a specific species that was not detected in previous IBD studies. These intestinal fungi alterations observed in UC patients might also lead to the development of specific fungal probiotics that lessens the inflammatory status resembling bacterial probiotics [31, 32].

Dectin-1 is the most important fungal receptor [33] and is related to the alteration of intestinal fungal composition during UC [6]. Furthermore, the evidence obtained by Takagawa et al*.* indicated that the overexpression of LRRK2 protein in mice bearing SNP *rs11564258* is related to increased Dectin-1 mediated pro-inflammatory cytokines [10]. In this regard, we observed higher mRNA expressions of Dectin-1 and LRRK2 in our UC patients compared with HS dissimilar to Iliev et al*.* murine study [6]. However, unlike our expectation, their expression was not affected by the presence of SNP *rs11564258*.

LRRK2 acting upon Dectin-1 receptor activation could also be elicited from the results. As explained by Tang et al*.* [34], the inhibition of Dectin-1 signaling could ameliorate colitis despite a few contradictory results reported in previous studies [35]. Given that only one report surveyed the association of LRRK2 with IBD and its influence on Dectin-1 mediated inflammation, this aspect of IBD needs to be clarified in future studies.

## Conclusions

In summary, we perceived a strong association between *rs11564258* polymorphism and UC susceptibility in addition to increased global fungal load detected in the active UC patients without being affected by this mutation. Therefore, we could not support the hypothesis of the association between mycobiota-genotype of *rs11564258* in UC patients. Our data also showed that LRRK2 mediates the activation of Dectin-1 signaling pathway in UC patients without being related to SNP *rs11564258*. However, we hypothesized that a decrease in fungal diversity and an excessive increase in the community of *C. albicans* may partially act in the genesis of UC by activating the LRRK2. More clinical investigations, particularly in larger populations or different ethnic groups, are required to support this conclusion.

## Data Availability

The datasets used and/or analyzed during the current study are available from the corresponding author on reasonable request.

## Abbreviations

IBD: Inflammatory bowel disease
CD: Crohn’s disease
UC: Ulcerative colitis
SNPs: Single-nucleotide polymorphisms
PRR: Pattern recognition receptor
CLEC7A: C-Type Lectin domain containing 7A
LRRK2: Leucine-rich repeat kinase 2
DCs: Dendritic cells
TNF-α: Tumor necrosis factor-alpha
HS: Healthy subjects
DAI: Disease activity index

## References

[1] Damaskos D, Kolios G (2008). Probiotics and prebiotics in inflammatory bowel disease: microflora 'on the scope'. Br J Clin Pharmacol.

[2] Lepage P, Seksik P, Sutren M, de la Cochetière MF, Jian R, Marteau P, Doré J (2005). Biodiversity of the mucosa-associated microbiota is stable along the distal digestive tract in healthy individuals and patients with IBD. Inflamm Bowel Dis.

[3] Sokol H, Seksik P (2010). The intestinal microbiota in inflammatory bowel diseases: time to connect with the host. Curr Opin Gastroenterol.

[4] Ott SJ, Kühbacher T, Musfeldt M, Rosenstiel P, Hellmig S, Rehman A, Drews O, Weichert W, Timmis KN, Schreiber S (2008). Fungi and inflammatory bowel diseases: alterations of composition and diversity. Scand J Gastroenterol.

[5] Jostins L, Ripke S, Weersma RK, Duerr RH, McGovern DP, Hui KY, Lee JC, Schumm LP, Sharma Y, Anderson CA (2012). Host-microbe interactions have shaped the genetic architecture of inflammatory bowel disease. Nature.

[6] Iliev ID, Funari VA, Taylor KD, Nguyen Q, Reyes CN, Strom SP, Brown J, Becker CA, Fleshner PR, Dubinsky M (2012). Interactions between commensal fungi and the C-type lectin receptor dectin-1 influence colitis. Science.

[7] Mahmoudi E, Mozhgani S-H, Sharifinejad N (2021). The role of mycobiota-genotype association in inflammatory bowel diseases: a narrative review. Gut Pathogens.

[8] Liu Z, Lee J, Krummey S, Lu W, Cai H, Lenardo MJ (2011). The kinase LRRK2 is a regulator of the transcription factor NFAT that modulates the severity of inflammatory bowel disease. Nat Immunol.

[9] Wallings R, Manzoni C, Bandopadhyay R (2015). Cellular processes associated with LRRK2 function and dysfunction. FEBS J.

[10] Takagawa T, Kitani A, Fuss I, Levine B, Brant SR, Peter I, Tajima M, Nakamura S, Strober W (2018). An increase in LRRK2 suppresses autophagy and enhances dectin-1-induced immunity in a mouse model of colitis. Sci Transl Med.

[11] Liu Z, Lenardo MJ (2012). The role of LRRK2 in inflammatory bowel disease. Cell Res.

[12] Wong AYW, Oikonomou V, Paolicelli G, De Luca A, Pariano M, Fric J, Tay HS, Ricciardi-Castagnoli P, Zelante T (2018). Leucine-rich repeat kinase 2 controls the Ca(2+)/nuclear factor of activated T cells/IL-2 pathway during Aspergillus non-canonical autophagy in dendritic cells. Front Immunol.

[13] Schmittgen TD, Livak KJ (2008). Analyzing real-time PCR data by the comparative CT method. Nat Protoc.

[14] Yamada Y, Makimura K, Merhendi H, Ueda K, Nishiyama Y, Yamaguchi H, Osumi M (2002). Comparison of different methods for extraction of mitochondrial DNA from human pathogenic yeasts. Jpn J Infect Dis.

[15] Schoch CL, Seifert KA, Huhndorf S, Robert V, Spouge JL, Levesque CA, Chen W, Fungal Barcoding C (2012). Nuclear ribosomal internal transcribed spacer (ITS) region as a universal DNA barcode marker for fungi. Proc Natl Acad Sci.

[16] Malekzadeh MM, Sima A, Alatab S, Sadeghi A, Daryani NE, Adibi P, Maleki I, Vossoughinia H, Fakheri H, Yazdanbod A (2019). Iranian Registry of Crohn's and Colitis: study profile of first nation-wide inflammatory bowel disease registry in Middle East. Intest Res.

[17] Pasvol TJ, Horsfall L, Bloom S, Segal AW, Sabin C, Field N, Rait G (2020). Incidence and prevalence of inflammatory bowel disease in UK primary care: a population-based cohort study. BMJ Open..

[18] Leonardi I, Li X, Semon A, Li D, Doron I, Putzel G, Bar A, Prieto D, Rescigno M, McGovern DPB (2018). CX3CR1+, mononuclear phagocytes control immunity to intestinal fungi. Science.

[19] Malik A, Sharma D, Malireddi RKS, Guy CS, Chang TC, Olsen SR, Neale G, Vogel P, Kanneganti TD (2018). SYK-CARD9 signaling axis promotes gut fungi-mediated inflammasome activation to restrict colitis and colon cancer. Immunity.

[20] Wang T, Pan D, Zhou Z, You Y, Jiang C, Zhao X, Lin X (2016). Dectin-3 deficiency promotes colitis sevelopment due to impaired antifungal innate immune responses in the gut. PLoS Pathogens.

[21] Franke A, McGovern DPB, Barrett JC, Wang K, Radford-Smith GL, Ahmad T, Lees CW, Balschun T, Lee J, Roberts R (2010). Genome-wide meta-analysis increases to 71 the number of confirmed Crohn's disease susceptibility loci. Nat Genet.

[22] Liguori G, Lamas B, Richard ML, Brandi G, da Costa G, Hoffmann TW, Di Simone MP, Calabrese C, Poggioli G, Langella P (2016). Fungal dysbiosis in mucosa-associated microbiota of Crohn's disease patients. J Crohns Colitis.

[23] Kalantar E, Assadi M, Pormazaheri H, Hatami S, Barari MA, Asgari E, Mahmoudi E, Kabir K, Amin Marashi SM (2014). Candida non albicans with a high amphotericin B resistance pattern causing Candidemia among cancer patients. Asian Pac J Cancer Prev.

[24] Standaert-Vitse A, Sendid B, Joossens M, François N, Vandewalle-El Khoury P, Branche J, Van Kruiningen H, Jouault T, Rutgeerts P, Gower-Rousseau C (2009). Candida albicans colonization and ASCA in familial Crohn's disease. Am J Gastroenterol.

[25] Hoarau G, Mukherjee PK, Gower-Rousseau C, Hager C, Chandra J, Retuerto MA, Neut C, Vermeire S, Clemente J, Colombel JF (2016). Bacteriome and mycobiome interactions underscore microbial dysbiosis in familial Crohn’s disease. mBio.

[26] Li Q, Wang C, Tang C, He Q, Li N, Li J (2014). Dysbiosis of gut fungal microbiota is associated with mucosal inflammation in crohn's disease. J Clin Gastroenterol.

[27] Sokol H, Leducq V, Aschard H, Pham HP, Jegou S, Landman C, Cohen D, Liguori G, Bourrier A, Nion-Larmurier I (2017). Fungal microbiota dysbiosis in IBD. Gut.

[28] Di Paola M, Rizzetto L, Stefanini I, Vitali F, Massi-Benedetti C, Tocci N, Romani L, Ramazzotti M, Lionetti P, De Filippo C, Cavalieri D (2020). Comparative immunophenotyping of *Saccharomyces cerevisiae* and *Candida* spp. strains from Crohn's disease patients and their interactions with the gut microbiome. J Transl Autoimmun.

[29] Mukhopadhya I, Hansen R, Meharg C, Thomson JM, Russell RK, Berry SH, El-Omar EM, Hold GL (2015). The fungal microbiota of de-novo paediatric inflammatory bowel disease. Microbes Infect.

[30] Nelson A, Stewart CJ, Kennedy NA, Lodge JK, Tremelling M, Probert CS, Parkes M, Mansfield JC, Smith DL, Hold GL (2020). The impact of NOD2 genetic variants on the gut mycobiota in Crohn's disease patients in remission and individuals without gastrointestinal inflammation. J Crohns Colitis.

[31] Asgari B, Kermanian F, Hedayat Yaghoobi M, Vaezi A, Soleimanifar F, Yaslianifard S (2020). The Anti-helicobacter pylori effects of *Lactobacillus acidophilus*, *L. plantarum*, and *L. rhamnosus* in stomach tissue of C57BL/6 Mice. Visc Med.

[32] Pakravan N, Kermanian F, Mahmoudi E (2019). Filtered Kombucha tea ameliorates the leaky gut syndrome in young and old mice model of colitis. Iran J Basic Med Sci.

[33] Taylor PR, Tsoni SV, Willment JA, Dennehy KM, Rosas M, Findon H, Haynes K, Steele C, Botto M, Gordon S, Brown GD (2007). Dectin-1 is required for beta-glucan recognition and control of fungal infection. Nat Immunol.

[34] Tang C, Kamiya T, Liu Y, Kadoki M, Kakuta S, Oshima K, Hattori M, Takeshita K, Kanai T, Saijo S (2015). Inhibition of dectin-1 signaling ameliorates colitis by inducing lactobacillus-mediated regulatory T cell expansion in the intestine. Cell Host Microbe.

[35] Heinsbroek SE, Oei A, Roelofs JJ, Dhawan S, te Velde A, Gordon S, de Jonge WJ (2012). Genetic deletion of dectin-1 does not affect the course of murine experimental colitis. BMC Gastroenterol.

